# ﻿First formal record of the feeding habits of Saileriolidae (Hemiptera, Heteroptera, Pentatomomorpha, Pentatomoidea), with redescription of *Bannacorishyalinus* (Schaefer & Ashlock, 1970), comb. nov. endemic to Vietnam

**DOI:** 10.3897/zookeys.1221.135026

**Published:** 2024-12-31

**Authors:** Jun Souma, Cuong Viet Canh Le, Thai-Hong Pham

**Affiliations:** 1 Shirakami Research Center for Environmental Sciences, Faculty of Agriculture and Life Science, Hirosaki University, Aomori, Japan Hirosaki University Aomori Japan; 2 Mientrung Institute for Scientific Research, Vietnam National Museum of Nature, Vietnam Academy of Science and Technology, Hue, Vietnam Vietnam Academy of Science and Technology Hue Vietnam; 3 Graduate School of Science and Technology, Vietnam Academy of Science and Technology, Hanoi, Vietnam Vietnam Academy of Science and Technology Hanoi Vietnam

**Keywords:** Host plant, phytophagous insect, rare species, Tingidae, Urostylididae

## Abstract

In the present study, the rare true bug *Bannacorishyalinus* (Schaefer & Ashlock, 1970), **comb. nov.** (Hemiptera, Heteroptera, Pentatomomorpha, Pentatomoidea, Saileriolidae), which is endemic to Vietnam, is redescribed and transferred from the genus *Saileriola* China & Slater, 1956 to the genus *Bannacoris* Hsiao, 1964 based on morphological characteristics. Adults and nymphs of this species congregate in groups of several individuals and suck sap from the abaxial side of the leaves of *Litsea* sp. (Lauraceae). They cause visible feeding damage on the adaxial side of *Litsea* leaves, similar to that caused by members of the heteropteran family Tingidae Laporte, 1832 (Cimicomorpha, Miroidea). The new knowledge of *B.hyalinus***comb. nov.** also represents the first formal record of the feeding habits of Saileriolidae China & Slater, 1956. An identification key to all four species of this family is provided.

## ﻿Introduction

The true bug family Saileriolidae China & Slater, 1956 (Hemiptera, Heteroptera, Pentatomomorpha, Pentatomoidea) comprises the following four species in three genera distributed in Asia: *Bannacorisarboreus* Hsiao, 1964 from China and Thailand; *Ruckesonavitrella* Schaefer & Ashlock, 1970 from Thailand; *Saileriolahyalina* Schaefer & Ashlock, 1970 from Vietnam; and *S.sandakanensis* China & Slater, 1956 from Malaysia (Borneo Island) (China and Slater 1956; Hsiao 1964; Schaefer and Ashlock 1970; Hsiao and Ching 1977; Rider 2006; Rider et al. 2018). This family was previously assigned to the pantatomoid family Urostylididae Dallas, 1851 (China and Slater 1956), but it was elevated to the family rank owing to the non-monophyly of Urostylididae (Grazia et al. 2008), which was supported by subsequent studies using the morphological characteristics of extant and fossil species (Yao et al. 2012, 2013). Nevertheless, recent studies partially or completely based on molecular data have recovered the sister relationship between Saileriolidae and Urostylididae, and these studies continued to treat Saileriolidae at the family rank (Wu et al. 2016; Zhou and Rédei 2020; Ye et al. 2022; Duan et al. 2023).

If it is reasonable to treat Saileriolidae as a family rank, there should be significant differences not only in morphological but also ecological characteristics between Saileriolidae and Urostylididae. Although the life history of Urostylididae has been reported in some Japanese species (Kobayashi and Tachikawa 2004; Kaiwa et al. 2014), the biological information of Saileriolidae is poorly understood (Rider et al. 2018; Schuh and Weirauch 2020). Current biological information on Saileriolidae based on the published literature is as follows: (i) several adults and nymphs of *R.vitrella* were collected from a “palm at the water margin” (Arecaceae), therefore the indeterminate palm could be a host plant for this species (cf. Schaefer and Ashlock 1970; Schuh and Weirauch 2020); (ii) the guts of adults and nymphs of *R.vitrella* contain green fragments similar to chloroplasts, suggesting that this species does not feed exclusively on sap but also ingests chloroplasts from leaves and/or stems (Schaefer and Ashlock 1970); and (iii) *B.arboreus* was collected from leaves of an indeterminate banana *Musa* sp. (Musaceae) (cf. Rider et al. 2018). Additionally, in the biological information not formally published, several adults and nymphs of this species have been observed congregating on the abaxial side of banana leaves (https://spain.inaturalist.org/taxa/1360964-Bannacoris-arboreus). In conclusion, more field surveys and formal publications on the relevant biological information are needed to elucidate the life history of Saileriolidae.

Meanwhile, the two known species of the genus *Saileriola* China & Slater, 1956 seem to be rare because only the old holotype is known (cf. China and Slater 1956; Schaefer and Ashlock 1970). In the original description, *R.vitrella* and *S.hyalina* were not compared with *B.arboreus*, and the general habitus of *S.hyalina* was not illustrated (cf. Schaefer and Ashlock 1970), making the identification key to the species incomplete and the identification of *S.hyalina* difficult. Therefore, a taxonomic study based on field surveys should be conducted to rediscover the two species of *Saileriola* and to provide an updated identification key to the four known species of the family, including *B.arboreus*.

Recently, the first author rediscovered *S.hyalina* in Bạch Mã National Park, Thừa Thiên Huế Province, Vietnam, with the help of the second and third authors. In addition, the first author observed the feeding habits of this species. Furthermore, *S.hyalina* is consistent with the diagnostic characters of the genus *Bannacoris* Hsiao, 1964 based on the examination of morphological characteristics by the first author. In the present study, we redescribe *S.hyalina* and propose a new combination, *Bannacorishyalinus* (Schaefer & Ashlock, 1970), comb. nov., which is transferred from *Saileriola* to *Bannacoris*. Moreover, we report on the biology of *B.hyalinus* comb. nov., providing the first formal record of the feeding habits of Saileriolidae. We also provide an identification key to the four known species of Saileriolidae.

## ﻿Materials and methods

The morphological characteristics of the specimens were observed, drawn, and measured using a stereoscopic microscope (SZX16; Olympus, Tokyo, Japan) equipped with an ocular grid. To examine the male and female genitalia, first, the terminalia was removed from the body after softening the specimens in hot water. The removed terminalia was then immersed in a hot 15% potassium hydroxide (KOH) solution for 5 min. For further observation, the paramere and phallus were immersed in 99% ethanol and removed from the genital capsule. Male and female genitalia were preserved in small polyethylene vials containing a 50% aqueous solution of glycerin. Male and female genitalia were observed after the angles were fixed with a gel (Museum Gel Clear; Ready America, California, USA) and placed on a microscope slide. The polyethylene vial was mounted on a pin with the respective specimens. The specimens were photographed using a digital camera (EOS 90D; Canon, Tokyo, Japan) equipped with a zoom lens (18–35 mm F1.8 DC HSM; SIGMA, Kanagawa, Japan) and a digital microscope (Dino-Lite Premier M; Opto Science, Tokyo, Japan). Photographs of living individuals and habitats were taken using a compact digital camera (Tough TG-6; Olympus, Tokyo, Japan) and a smartphone (iPhone 14; Apple, California, USA), respectively. The image stacks of the specimens were processed using a Zerene Stacker (Zerene Systems, Richland, WA, USA). All illustrations and photographs were edited using Adobe Photoshop 2024 v. 25.11. Morphological terms were generally assigned according to Tsai et al. (2011).

The specimens examined in this study have been deposited at the Vietnam National Museum of Nature, Hanoi, Vietnam (**VNMN**).

The species distribution map was created and edited using Adobe Photoshop, and geographic coordinates were obtained from Google Maps (https://www.google.co.jp/maps).

## ﻿Results

### ﻿Taxonomy

#### ﻿Genus Bannacoris

Taxon classificationAnimaliaHemipteraSaileriolidae

Hsiao, 1964

336ACBC9-E75C-5D31-BA69-80E6B0DEB2B6

Bannacoris Hsiao, 1964: 283. Type species by original designation: Bannacorisarboreus Hsiao, 1964.

##### Diagnosis.

*Bannacoris* can be distinguished from the two other known saileriolid genera, *Ruckesona* Schaefer & Ashlock, 1970 and *Saileriola* China & Slater, 1956, by a combination of the following characters: head with a median sulcus on vertex (without a median sulcus on vertex in *Ruckesona*); compound eye separated from anterior margin of pronotum (close to anterior margin of pronotum in *Ruckesona* and *Saileriola*); a pair of ocelli closer together than a diameter of ocellus (separated by more than 3.0 times of a diameter of ocellus in *Ruckesona*); antennomere I more than 1.5 times as long as antennomere II (less than 1.5 times in *Ruckesona*); lateral margin of pronotum serrate in anterior part (nearly straight in *Saileriola*), without distinct spine (with two distinct spines in *Saileriola*); and corium of forewing mostly punctate (punctate only along claval and median furrows in *Saileriola*).

##### Remarks.

The following characters in the original description are the diagnostic characters of the family Saileriolidae, partly including misinterpretation, and are unable to distinguish this genus from other saileriolid genera (Hsiao 1964; Schaefer and Ashlock 1970; Rider et al. 2018; present study): ostiole of metathoracic scent gland quite small (absent in original description); peritreme absent; and tarsi three-segmented (two-segmented in original description). Additionally, hindwing venation, which differs between *Ruckesona* and *Saileriola*, was not considered in the present study because of the lack of a detailed description of the type species of *Bannacoris*, *B.arboreus* (cf. Hsiao 1964). Thus, based on the original descriptions of the three known saileriolid genera (China and Slater 1956; Hsiao 1964; Schaefer and Ashlock 1970) and the first author’s examination, we provisionally redefine the morphological characteristics shared by Bannacoris species, as described in the Diagnosis section above, and propose a new combination, *Bannacorishyalinus* (Schaefer & Ashlock, 1970), comb. nov., which is transferred from *Saileriola* to *Bannacoris*. In conclusion, the genus *Bannacoris* comprises two species, *B.arboreus* from China and Thailand and *B.hyalinus* comb. nov. from Vietnam (Hsiao 1964; Schaefer and Ashlock 1970; Rider et al. 2018; present study).

#### ﻿ Bannacoris hyalinus

Taxon classificationAnimaliaHemipteraSaileriolidae

(Schaefer & Ashlock, 1970) comb. nov.

E74F79CF-237F-5458-A096-E650B3BCDD0E

**Figure 1.**
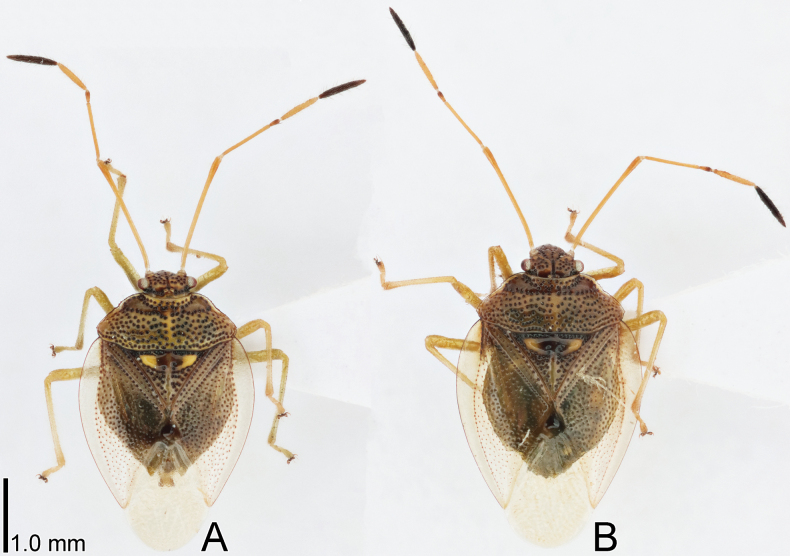
Dorsal habitus of *Bannacorishyalinus* comb. nov. from Vietnam. **A** male **B** female.

**Figure 2.**
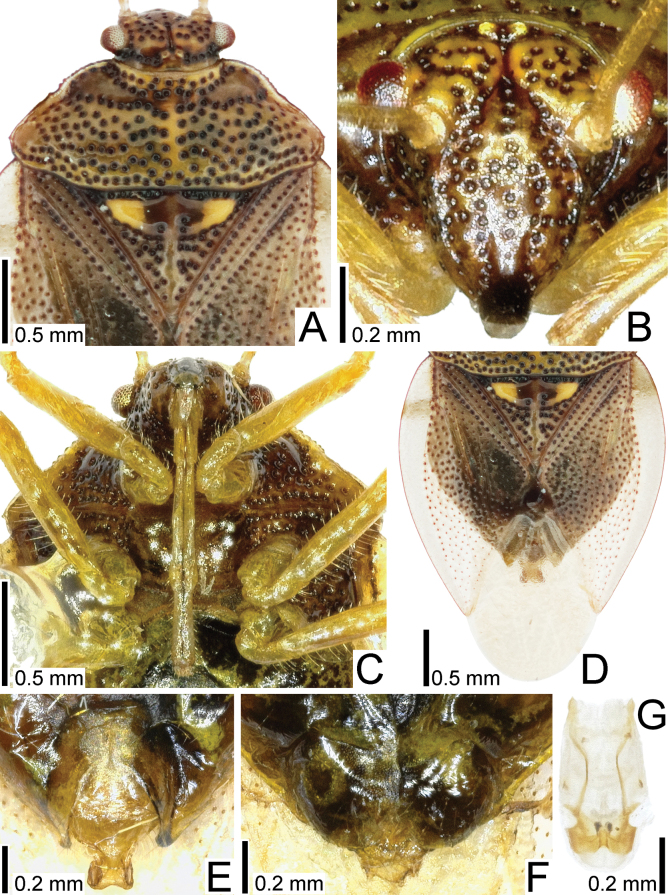
Detailed morphological images of *Bannacorishyalinus* comb. nov. from Vietnam **A** head, pronotum, and scutellum, dorsal view **B** head, cranial view **C** head and thorax, ventral view **D** forewing, dorsal view **E** male terminalia, ventral view **F** female terminalia, ventral view **G** phallus, ventral view.

**Figure 3.**
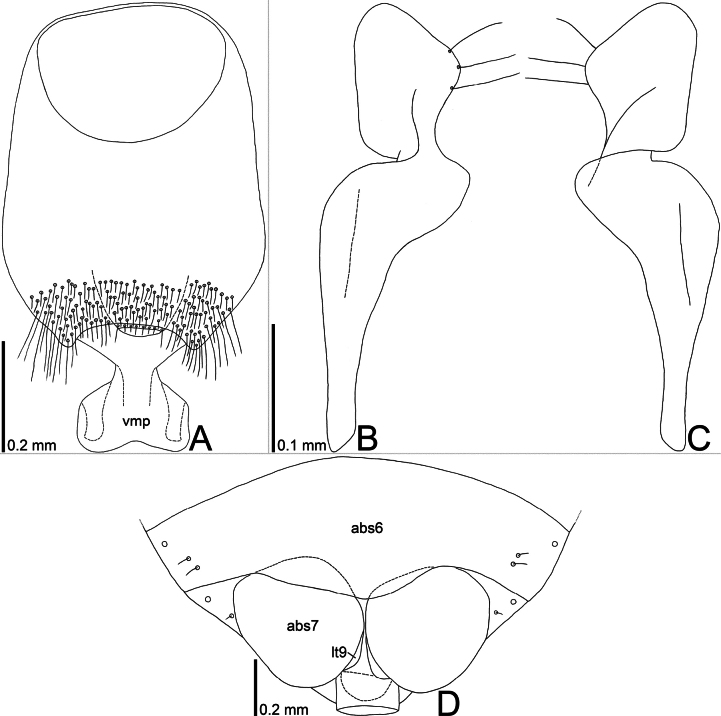
Line drawings of *Bannacorishyalinus* comb. nov. from Vietnam **A** genital capsule, dorsal view **B** paramere, dorsal view **C** paramere ventral view **D** female terminalia, ventral view. Abbreviations: abs6, abs7, abdominal sternites VI, VII; lt9, laterotergite IX; vmp, ventromedian process.

**Figure 4.**
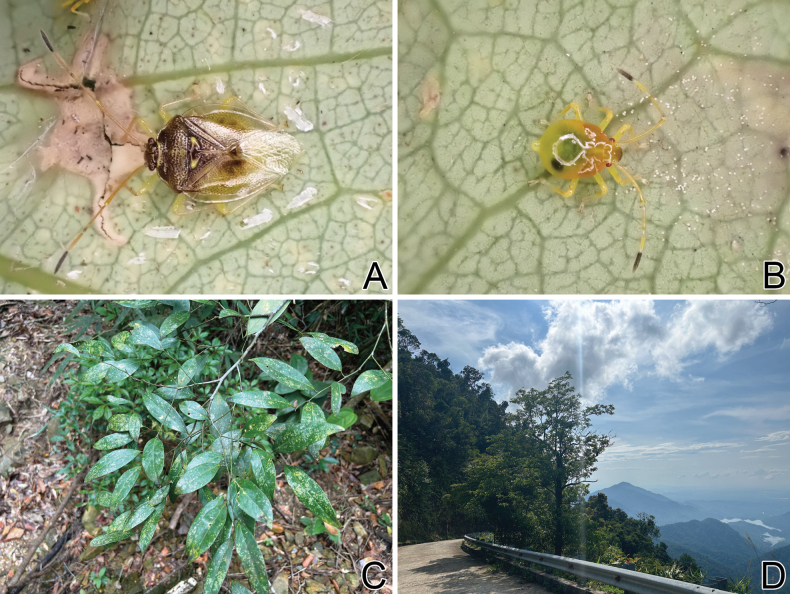
Photographs regarding *Bannacorishyalinus* comb. nov. from Bạch Mã National Park, Thừa Thiên Huế Province, Vietnam **A** living adult **B** living nymph **C** host plant (*Litsea* sp.) **D** surrounding habitat.

Figs 1A, B , 2A–G , 3A–D , 4A, B

Saileriola hyalina Schaefer & Ashlock, 1970: 631. Holotype: ♂, Vietnam: 7 km SE of Dilinh (Djiring), 990 m [= Lâm Đồng Province, Di Linh District, Bảo Thuận?]; Bernice P. Bishop Museum, Honolulu, Hawaii, USA.

##### Material examined.

**Non-types** (5 ♂♂ 4 ♀♀, VNMN): Vietnam • Thừa Thiên Huế Province, Phú Lộc District, Bạch Mã National Park, Lộc Trì, Đường mòn Đỗ Quyên; 16°11'34"N, 107°50'52"E; 6.vi.2024; leg. J. Souma.

##### Diagnosis.

*Bannacorishyalinus* comb. nov. can be distinguished from the only other congener, *B.arboreus*, by the following characters: head, pronotum, and scutellum mostly yellowish brown (Figs 1A, B, 2A) (mostly reddish to dark brown in *B.arboreus*); corium of forewing mostly hyaline (Fig. 2D) (reddish to dark brown in middle part in *B.arboreus*), not punctate in an area enclosed by Sc (subcostal) vein and medial furrow (entirely punctate in *B.arboreus*); dorsolateral process of genital capsule undeveloped (Figs 2E, 3A) (protruding posteriad in *B.arboreus*); and ventromedian process concave in posterior margin (gently curved outward in *B.arboreus*).

##### Redescription.

Most parts of head, antennomeres I–IV, pronotum except for anterior and lateral margins, ventral surface of thoracic parts, most parts of scutellum, legs except for claws, and abdomen yellowish brown; antennomere V, compound eye, ocellus, anterior margin of scutellum, Sc (subcostal) vein of forewing, claws, and punctures on body dark brown; anterior and lateral margins of pronotum, and outer part of semi-elliptical ridge in anteromedial part of scutellum whitish brown; forewing except for Sc vein and punctures hyaline; setae on body yellowish (Figs 1A, B, 2A–F, 4A, B).

Body (Fig. 1A, B) ovate, 1.5–1.6 times as long as maximum width across abdomen. Head (Fig. 2A–C) declivent, mostly punctate, longer than maximum width across compound eyes in cranial view, with a median sulcus on vertex, sparsely bearing minute setae. Antenniferous tubercle annulate, placed anterior to compound eye. Clypeus distinctly surpassing mandibular plate at both apices. Compound eye round, separated from anterior margin of pronotum. A pair of ocelli placed along midline of vertex, closer together than a diameter of ocellus. Antenna smooth on surface; antennomere I longest among antennomeres, more than 1.5 times as long as antennomere II, bearing minute setae throughout its length; antennomere II longer than antennomere IV, bearing minute setae throughout its length; antennomere III shortest among antennomeres, bearing minute setae throughout its length; antennomere IV as long as antennomere V, bearing minute and long setae throughout its length; antennomere V bearing minute and long setae throughout its length. Labium reaching anterior part of abdominal sternite III. Buccula semi-elliptical in lateral view, highest in middle part.

Thorax (Figs 1A, B, 2A, C) mostly punctate. Pronotum trapezoidal in dorsal view, shorter than its maximum width, punctate except for callus; lateral margin serrate in anterior part, without distinct spine, bearing minute setae throughout its length; humeral angle rounded. Scutellum triangular, shorter than its maximum width, semi-elliptically raised in anteromedial part, punctate except for midline and semi-elliptical ridge. Forewing (Fig. 2D) oblong; anterior margin gently curved outward; clavus shorter than membrane, with 2 rows of punctures throughout its length; corium punctate except in an area enclosed by Sc vein and medial furrow, bearing minute setae in anterior part; membrane provided with several indistinct longitudinal veins; Sc vein, basal part of Cu (cubital) vein, and claval and medial furrows distinct. Epimera and episterna punctate except metepimeron. Sterna smooth on surface. Legs smooth on surface; femora and tibiae cylindrical, bearing setae throughout their length.

Abdomen (Figs 1A, B, 2D–F) longer than combined length of head and pronotum; posterior margin of sternite VI concave in male, undulate in female; sternite VII concave in posterior margin of male, with a longitudinal cleft in female. Genital capsule (Fig. 3A) elliptical in dorsal and ventral views, smooth on surface, bearing setae in posterior part; lateral margin gently curved outward; dorsolateral process undeveloped; ventromedian process widened apically, concave in posterior margin. Paramere (Fig. 3B, C) elongate; crown widened apically, bearing three setae from cuticular sockets along outer margin of dorsum; neck constricted; stem widened apically. Phallus (Fig. 2G) oblong; basal plate and phallotheca coriaceous; conjunctiva with two pairs of sclerites. Female terminalia (Fig. 3D) semicircular in ventral view, protruding posteriad in middle part; laterotergite VIII reduced; valvifer VIII reduced; laterotergite IX rounded in outer margin.

Measurements (male: *n* = 5; female: *n* = 4). Body length with forewing 3.5–3.8 mm in male and 3.9–4.0 mm in female, maximum width across forewings 2.3–2.4 mm in male and 2.5 mm in female; head length in cranial view 0.9 mm in both sexes, maximum width across compound eyes 0.8 mm in both sexes; length of antennomeres I–V in both sexes 1.5 mm, 0.8 mm, 0.2 mm, 0.6 mm, and 0.6 mm, respectively; length of labial segments I–IV in both sexes 0.3 mm, 0.3 mm, 0.3 mm, and 0.4 mm, respectively; pronotum length 0.7 mm in both sexes, maximum width 1.8 mm in male and 1.9–2.0 mm in female; scutellum length 0.8 mm in male and 0.9 mm in female, maximum width 1.0 mm in male and 1.1 mm in female; forewing length 2.8–2.9 mm in male and 3.0–3.1 mm in female, maximum width 1.2 mm in male and 1.3 mm in female.

##### Remarks.

The nine specimens recorded above (Fig. 1A, B) matched well with the original description and illustrations of *Bannacorishyalinus* comb. nov. (Schaefer and Ashlock 1970) in terms of morphological characteristics, especially the structure of the head (Fig. 2B) and the shape of the genital capsule (Figs 2E, 3A) and paramere (Fig. 3B, C). Therefore, we identified the specimens studied as *B.hyalinus* comb. nov. and redescribed this species in the above section.

In the original description (Schaefer and Ashlock 1970), *B.hyalinus* comb. nov. was not compared with the only other congener, *B.arboreus*, making the identification of the two species difficult. However, based on the comparison among the nine specimens of *B.hyalinus* comb. nov. and the illustrations (Hsiao 1964; Hsiao and Ching 1977), photographs (Hsiao and Ching 1977; Rider et al. 2018; https://spain.inaturalist.org/taxa/1360964-Bannacoris-arboreus), and original description (Hsiao 1964) of *B.arboreus*, the five characters described in the Diagnosis section above were recognized to easily differentiate *B.hyalinus* comb. nov. from *B.arboreus*.

##### Distribution.

**Figure 5.**
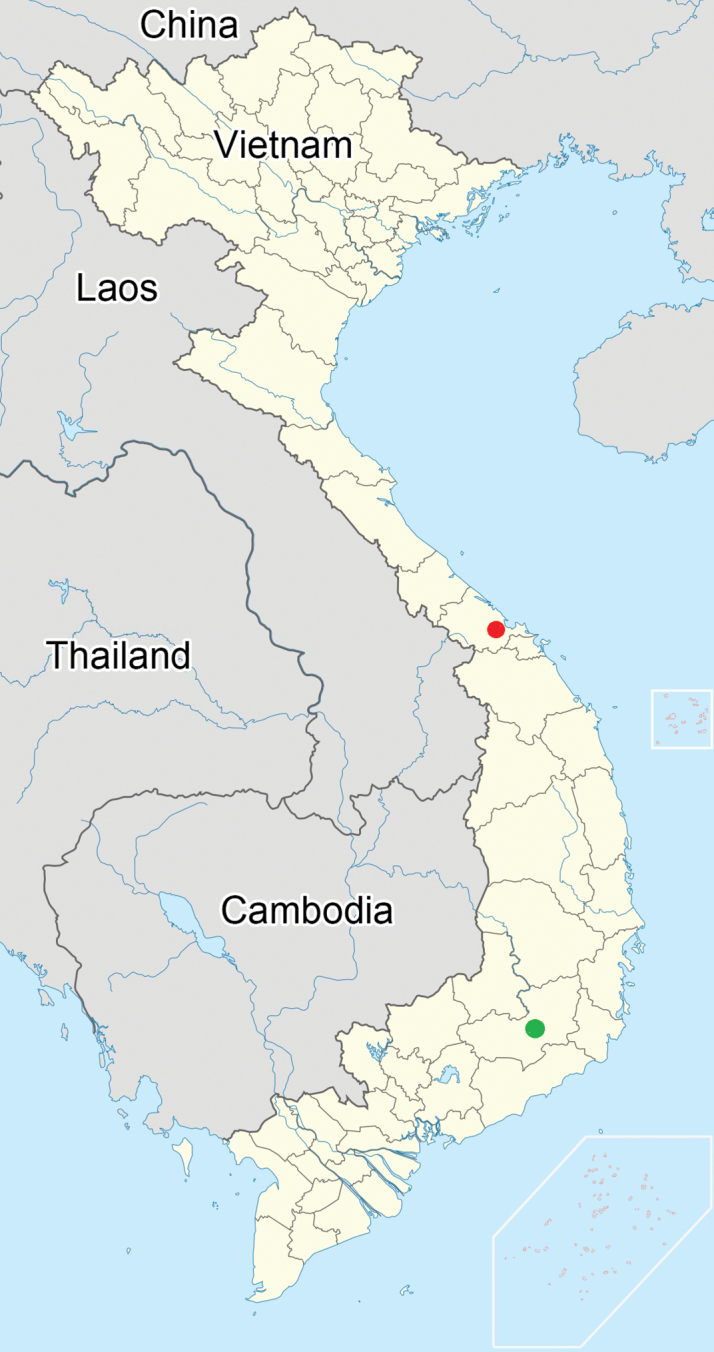
Collection sites of *Bannacorishyalinus* comb. nov.: red circle = new record; green circle = known record.

Vietnam (Thừa Thiên Huế Province, Lâm Đồng Province) (Fig. 5) (Schaefer and Ashlock 1970; present study).

##### Host plant.

Adults and nymphs of *Bannacorishyalinus* comb. nov. were observed to congregate in groups of several on the abaxial side of leaves of *Litsea* sp. (Lauraceae) (Fig. 4C) in Bạch Mã National Park, Thừa Thiên Huế Province, Vietnam (Fig. 4D), by the first author. In addition, nymphs were observed sucking sap from the abaxial side of the leaves of this lauraceous tree in captivity. Thus, *Litsea* sp. is considered the host plant of *B.hyalinus* comb. nov., the biological information of which was unknown in the original description (Schaefer and Ashlock 1970).

The adaxial side of the leaves apparently damaged by this saileriolid species was irregularly yellowed in the field and in captivity, suggesting the possibility that *B.hyalinus* comb. nov. feeds on leaf chlorophyll.

##### Bionomics.

*Bannacorishyalinus* comb. nov. inhabits evergreen broad-leaved forests in the mountainous areas of Vietnam with a subtropical climate.

Adults were collected in May 1960 and June 2024 (Schaefer and Ashlock 1970; present study), and nymphs were observed in June 2024 (present study).

### ﻿Key to the species of the family Saileriolidae

Modified after the key provided by Schaefer and Ashlock (1970).

**Table:** 

1	Head without a median sulcus on vertex; a pair of ocelli separated by more than 3.0 times of a diameter of ocellus; antennomere I less than 1.5 times as long as antennomere II	***Ruckesonavitrella* Schaefer & Ashlock, 1970**
–	Head with a median sulcus on vertex (Figs 1A, B, 2A–C); a pair of ocelli closer together than a diameter of ocellus; antennomere I more than 1.5 times as long as antennomere II	**2**
2	Compound eye close to anterior margin of pronotum; lateral margin of pronotum nearly straight in anterior part, with two distinct spines; corium of forewing punctate only along claval and median furrows	***Saileriolasandakanensis* China & Slater, 1956**
–	Compound eye separated from anterior margin of pronotum (Figs 1A, B, 2A); lateral margin of pronotum serrate in anterior part, without distinct spine; corium of forewing mostly punctate (Fig. 2D)	**3**
3	Head, pronotum, and scutellum mostly yellowish brown (Figs 1A, B, 2A–C); corium of forewing mostly hyaline (Fig. 2D), not punctate in an area enclosed by Sc (subcostal) vein and medial furrow	***Bannacorishyalinus* (Schaefer & Ashlock, 1970), comb. nov.**
–	Head, pronotum, and scutellum mostly reddish to dark brown; corium of forewing reddish to dark brown in middle part, entirely punctate	***B* . *arboreus* Hsiao, 1964**

## ﻿Discussion

In this study, the feeding habits of Saileriolidae was formally reported for the first time based on observations of *Bannacorishyalinus* comb. nov. endemic to Vietnam. This saileriolid species congregates in groups of several individuals and sucks sap from the abaxial side of the leaves of *Litsea* sp. (Fig. 4C). According to previous knowledge, *B.arboreus* and *Ruckesonavitrella* were collected from the leaves of indeterminate banana *Musa* sp. (Musaceae) and indeterminate palm (Arecaceae), respectively (cf. Schaefer and Ashlock 1970; Rider et al. 2018), and several adults and nymphs of *B.arboreus* were observed congregating on the abaxial side of banana leaves (https://spain.inaturalist.org/taxa/1360964-Bannacoris-arboreus). Therefore, members of Saileriolidae may generally suck sap from the abaxial side of the leaves. Moreover, the adaxial side of the leaves apparently damaged by *B.hyalinus* comb. nov. was irregularly yellowed, and the guts of adults and nymphs of *R.vitrella* contained green fragments similar to chloroplasts (Schaefer and Ashlock 1970), suggesting that members of Saileriolidae feed on leaf chlorophyll, as speculated in an earlier study (Schaefer and Ashlock 1970).

The feeding habits of Saileriolidae and folivorous taxa of the heteropteran family Tingidae Laporte, 1832 (Cimicomorpha, Miroidea), which feed on leaf chlorophyll, are similar in that in groups of several individuals congregate and suck sap on the abaxial side of the leaves, causing irregular yellowing on the adaxial side (cf. Ishihara and Kawai 1981; Yasunaga et al. 1993; Schuh and Weirauch 2020; Souma 2022). Nevertheless, since Saileriolidae and Tingidae belong to the infraorders Pentatomomorpha Leston, Pendergrast & Southwood, 1954 and Cimicomorpha Leston, Pendergrast & Southwood, 1954, respectively, and are distantly related (Schuh and Weirauch 2020; Ye et al. 2022), the similarity in feeding habits does not reflect phylogenetic relationships.

Meanwhile, the feeding habits of the pantatomoid family Urostylididae, which is a sister group of Saileriolidae (Wu et al. 2016; Zhou and Rédei 2020; Ye et al. 2022; Duan et al. 2023), differs from that of Saileriolidae as follows: (i) the first instar nymphs suck from a jelly-like substance enclosing the egg mass and develop into the second or third instar nymphs (Kobayashi and Tachikawa 2004; Kaiwa et al. 2014); (ii) the second or third instar nymphs to adults suck sap from various parts of host plants such as sprouts, shoots, leaves, and young fruits (Kobayashi and Tachikawa 2004); and (iii) adults and nymphs are not known to congregate on the abaxial side of leaves and cause visible feeding damage on the adaxial side. Furthermore, the photograph of the indeterminate egg mass, probably from *B.arboreus*, is not enclosed by a jelly-like substance (https://spain.inaturalist.org/taxa/1360964-Bannacoris-arboreus). Thus, young nymphs of this saileriolid species may suck sap from the host plant. In conclusion, although the life history of Saileriolidae is still not completely known, the differences in the feeding habits of Saileriolidae and Urostylididae possibly support the rationality of the treatment of previous studies that the former is not a subfamily of the latter but an independent family (Grazia et al. 2008; Yao et al. 2012, 2013; Wu et al. 2016; Zhou and Rédei 2020; Ye et al. 2022; Duan et al. 2023).

## Supplementary Material

XML Treatment for Bannacoris

XML Treatment for Bannacoris hyalinus

## ﻿Additional information

### Author ORCIDs

Jun Souma https://orcid.org/0000-0002-2238-5015

Cuong Viet Canh Le https://orcid.org/0000-0002-1430-6305

Thai-Hong Pham https://orcid.org/0000-0002-4763-3679

### Data availability

All of the data that support the findings of this study are available in the main text.
